# cudaMMC: GPU-enhanced multiscale Monte Carlo chromatin 3D modelling

**DOI:** 10.1093/bioinformatics/btad588

**Published:** 2023-09-29

**Authors:** Michal Wlasnowolski, Pawel Grabowski, Damian Roszczyk, Krzysztof Kaczmarski, Dariusz Plewczynski

**Affiliations:** Laboratory of Bioinformatics and Computational Genomics, Faculty of Mathematics and Information Science, Warsaw University of Technology, Warsaw 00-662, Poland; Laboratory of Functional and Structural Genomics, Centre of New Technologies, University of Warsaw, Warsaw 02-097, Poland; Department of Information Processing Systems, Faculty of Mathematics and Information Science, Warsaw University of Technology, Warsaw 00-662, Poland; Department of Information Processing Systems, Faculty of Mathematics and Information Science, Warsaw University of Technology, Warsaw 00-662, Poland; Department of Information Processing Systems, Faculty of Mathematics and Information Science, Warsaw University of Technology, Warsaw 00-662, Poland; Laboratory of Bioinformatics and Computational Genomics, Faculty of Mathematics and Information Science, Warsaw University of Technology, Warsaw 00-662, Poland; Laboratory of Functional and Structural Genomics, Centre of New Technologies, University of Warsaw, Warsaw 02-097, Poland

## Abstract

**Motivation:**

Investigating the 3D structure of chromatin provides new insights into transcriptional regulation. With the evolution of 3C next-generation sequencing methods like ChiA-PET and Hi-C, the surge in data volume has highlighted the need for more efficient chromatin spatial modelling algorithms. This study introduces the cudaMMC method, based on the Simulated Annealing Monte Carlo approach and enhanced by GPU-accelerated computing, to efficiently generate ensembles of chromatin 3D structures.

**Results:**

The cudaMMC calculations demonstrate significantly faster performance with better stability compared to our previous method on the same workstation. cudaMMC also substantially reduces the computation time required for generating ensembles of large chromatin models, making it an invaluable tool for studying chromatin spatial conformation.

**Availability and implementation:**

Open-source software and manual and sample data are freely available on https://github.com/SFGLab/cudaMMC.

## 1 Introduction

In recent years, the development of high-throughput sequencing methods and chromosome conformation capture (3C) technology has shown the significant influence of chromatin spatial conformation on genetic expression. Dynamic changes in the 3D structure at various levels of DNA spatial organization: from the entire chromosomes, chromosomal territories, domains (TAD, CCDs), or single chromatin loops, affect the transcription level of individual genes [reviewed in Chiliński *et al.* (2022)]. There is plenty of evidence that rearrangements of the spatial chromatin structure could alter gene expression. These changes may occur dynamically in the cell environment induced by heat stress or cell differentiation (Ray *et al.* 2019, Pei *et al.* 2022), as well as caused by genetic mutations, viruses infections, Structural Variations, and DNA methylations (Heinz *et al.* 2018, Lazniewski *et al.* 2019, Sadowski *et al.* 2019, Wlasnowolski *et al.* 2020). To investigate these phenomena, various algorithms for generating chromatin 3D structures have been developed. Notable examples include the methods described in Kadlof *et al.* (2020) and Di Pierro *et al.* (2016). For a broader overview, see reviews in Lin *et al.* (2019), Belokopytova and Fishman (2020), and Madsen-Østerbye *et al.* (2022). Among other chromatin 3D structure modelling methods, one is the *3D-GNOME* approach (Szałaj *et al.* 2016). 3D-GNOME enables the generation of individual 3D chromatin models and ensembles of model sets using the simulated annealing Monte Carlo approach. Modelling is performed based on a map of chromatin contacts mediated by specific proteins such as CTCF or RNAPII, which play a significant role in chromatin spatial organization. This modelling technique takes into account the hierarchical organization of the chromatin structure, from chromosome positioning to chromatin domains (TADs, CCDs), and down to individual chromatin loop shapes. Due to the substantial development of 3C techniques, the data volumes of the chromatin contacts have increased significantly. This substantially increases the computation time needed to model the ensemble of large chromosomal structures, making it less useful for genome-wide modelling. To address this challenge, we developed cudaMMC, which extended 3D-GNOME by implementing parallel acceleration using GPUs, resulting in a significant speed-up of calculations while preserving modelling quality. This improvement was essential for the calculation of spatial distance distribution between specific genomic loci, for which purpose we applied cudaMMC recently in the 3D-GNOME web server update (Wlasnowolski *et al.* 2023) for statistical analysis of changes of distances between gene promoters and enhancers. In addition, we added a new option for the output file format, mmCIF, which enables the presentation and analysis of chromatin 3D models using commonly used molecular 3D viewers, such as UCSF Chimera (Pettersen *et al.* 2004).

## 2 Materials and methods

### 2.1 *3D-GNOME—*CPU-oriented approach

The *3D-GNOME* method consists of two stages. In the first one, a chromosome is divided into several regions based on PET cluster interaction patterns so that each region can correspond to one topological domain. Next, Monte Carlo simulated annealing is used to position beads to minimize energy function, considering the distance between beads corresponding to different regions. The second stage models the position of chromatin anchors within each domain independently based on energy terms. Next, chromatin loops are modelled by inserting sub-anchor beads between adjacent anchors, wherefore their position and shape are set using minimized energy function again.

### 2.2 cudaMMC approach GPU-extended

The method speed-up comes from a massive parallel search in the configuration space. The ability to run thousands of independent threads effectively at the same time gives the GPU an advantage over CPU processors for tasks which can be divided into numerous smaller parts. Parallelization will be more productive if sequential parts of the algorithm and synchronization points are minimized or completely removed. In the case of simulated annealing, the balance between parallel and sequential computations is hard to achieve since global energy is calculated after local bead matching improvement. However, if we assume that local configuration changes have an impact only in a limited surrounding area, we can propose an algorithm which solves the problem in parallel sustaining the global fitting at a level not worse than the sequential algorithm.

**Figure 1.**
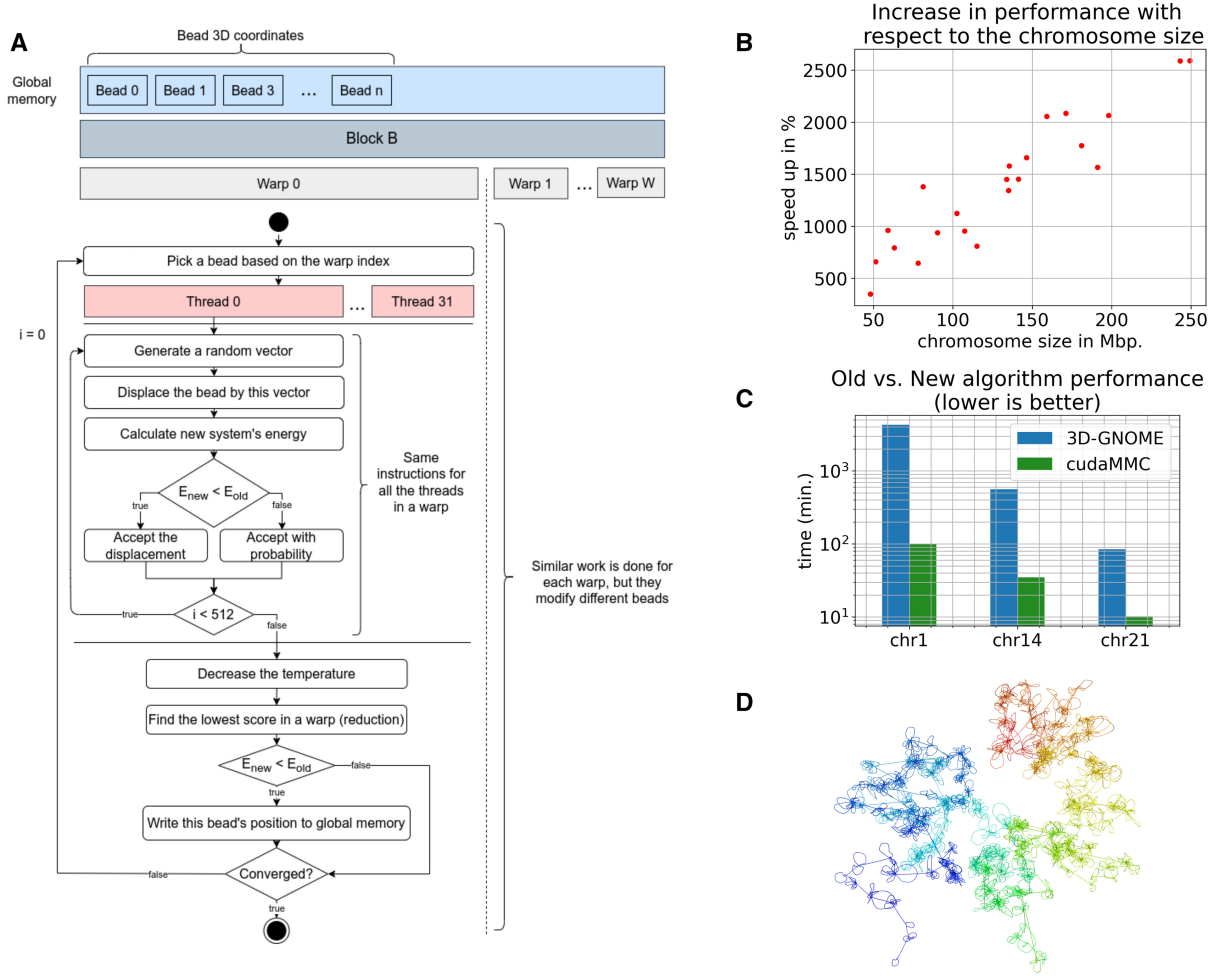
(A) Pipeline of the CUDA-based parallel simulated annealing algorithm for chromatin 3D structure modelling. (B) Speedup of the parallel algorithm in relation to chromosome size. Results shown are averages from 10 modelling runs using both methods. (C) Performance comparison between the 3D-GNOME (old) and cudaMMC (new) algorithms, based on generating ensembles of 100 models for selected chromosomes. Results shown are averages from three modelling runs for each method. (D) Full chromosomal chromatin 3D structure of chr1, based on CTCF ChIA-PET chromatin interactions from the GM12878 cell line mapped to GRCh37.

The main proposal is based on the idea that a parallel search for a better fitting is done within a so-called threads warp, a group of 32 cooperative threads, while energy function is evaluated globally using temporal results from other warps if only possible (Fig. 1A). A single warp optimizes a single bead using 32 threads to try random moves of that bead iteratively an arbitrarily selected number of times. Each thread chooses its best solution through the entire process, and then the parallel reduction is used to choose the best solution from all the warp. The final improvement proposal is accepted globally if it gives global improvement calculated in a given time from currently available improvements in other beads. Local improvement may not be positive if we consider other beads being modified at the same time. The overall process avoids global synchronization points, and experiments proved that the numerical stability of the method is not worse than the sequential one. The parallel method stop condition depends on the energy calculated and the number of improvement trials. In case of no possibilities of improvement, the method will finish.

### 2.3 Chromatin interaction data

We compared cudaMMC and *3D-GNOME* algorithms on *long-read* ChIA-PET CTCF chromatin interaction data for the GM12878 cell line mapped on GRCh37, for which data *3D-GNOME* was designed (ST1). We have also performed modelling tests using cudaMMC on *in situ* ChIA-PET data for the GM12878 cell line mapped on GRCh38 (ST2). We omitted the *3D-GNOME* performance testing on GRCh38 data because of the excessive computational time required caused by the massive increase of data gained by the new ChIA-PET method.

## 3 Comparison of performance between cudaMMC and 3D-GNOME

We compared the performance of the cudaMMC and 3D-GNOME methods on a workstation with a NVIDIA Pascal GPU architecture and an Intel Core i9-7920X CPU. The version of 3D-GNOME used for this benchmark was sourced directly from its official BitBucket repository (https://bitbucket.org/3dome/3dgnome) and represents the latest available version. The comparison was conducted using datasets of varying sizes. The cudaMMC algorithm achieved a speed-up ranging from 3× to 25× for single chromosome modelling, with the speed-up dependent on the chromosome size (Fig. 1B, Supplementary Fig. S1, and Supplementary Table ST1). The benchmark consisted of 10 modelling runs for each chromosome using both methods, and the comparative analysis was based on average execution times. The biggest advantage of the algorithm speed-up was observed for generating ensembles of models. Using GRCh37 data, we performed ensembles of 100 models each using both methods. The cudaMMC algorithm decreased computation time from ∼85 to ∼10 min for chr21, from ∼9 h to 35 min for chr14, and from ∼3 days to 1.5 h for chr1 (Fig. 1C and Supplementary Table ST3). Furthermore, we tested the performance of the cudaMMC algorithm for whole chromosomal modelling using in situ ChIA-PET data mapped to GRCh38 (ST4). On average, modelling chr1 took 50 min, chr14 took ∼17 min, and chr21 took 4.5 min. The benchmark consisted of three modelling runs. For the GRCh38 datasets, the 3D-GNOME algorithm was unable to complete the modelling process within a reasonable time frame. These results were obtained on Pascal architecture, but cudaMMC might also be configured on Turing or Amper NVIDIA devices perhaps with even better results.

In evaluating the consistency of execution times across methods, we performed the coefficient of variation (CoV) analysis. For 3D-GNOME, the CoVs were 32.05% for chr1, 19.79% for chr14, and 6.45% for chr21. In contrast, cudaMMC displayed significantly more consistent results with CoVs of 2.48% for chr1, 1.24% for chr14, and 2.01% for chr21. This underscores cudaMMC’s enhanced stability across different chromosomes (Supplementary Fig. S2). Lastly, we assessed the quality of cudaMMC modelling by comparing the similarity matrices within an ensemble of 100 models of chr21 between both methods (Supplementary Fig. S3). The distributions displayed in the density plots for both methods are nearly identical, with a difference in median values of <1%.

We added a tool for converting output into mmCIF format to make it more accessible for common usage. We chose this format instead of PDB because it can represent a higher number of beads for one structure, which is necessary for high-resolution whole chromosome models (Fig. 1D). Overall, our results show that the cudaMMC algorithm can significantly improve performance for 3D modelling of chromatin structures, making it a valuable tool for researchers in this field.

## 4 Conclusions

In the cudaMMC method, parallelizing calculations on the GPU enabled a massive reduction in computation time compared to the previous CPU-oriented approach of 3D-GNOME. This speed-up in calculations enables the generation of a high number of model ensembles in a reasonable time frame, thereby offering an opportunity for statistical analysis. For example, as part of the update to the 3D-GNOME web server, we have recently added a tool for statistical analysis of ensembles, which calculates changes in distances between gene promoters and enhancers of two model ensembles that differ in chromatin loop patterns caused by Structure Variants. To facilitate this, as a part of the 3D-GNOME 3.0 web server update (Wlasnowolski *et al.* 2023), we have set up the cudaMMC software on the NVIDIA DGX A100 cluster, enabling the running of multiple modelling tasks simultaneously on several GPU cards and facilitating analysis based on new, large datasets of spatial chromatin data. We believe that cudaMMC, the GPU-accelerated 3D-GNOME modelling engine, will significantly enhance scientists' investigations into various aspects of chromatin 3D structures.

## Supplementary Material

btad588_Supplementary_Data

## Data Availability

The data underlying this article used for benchmarking will be shared on reasonable request to the corresponding author.
