# Supplementary material for: cudaMMC: GPU-enhanced multiscale Monte Carlo chromatin 3D modelling
Source: Bioinformatics. 2023 Sep 29;39(10):btad588. doi: 10.1093/bioinformatics/btad588 (PMC10568367; doi:10.1093/bioinformatics/btad588)
Supplement: btad588_Supplementary_Data [file btad588_supplementary_data.docx]

**Supplementary information:
cudaMMC - GPU-enhanced Multiscale Monte Carlo Chromatin 3D Modelling**

Michał Wlasnowolski^1,2,♱^, Paweł Grabowski^1,♱^, Damian Roszczyk^1,♱^,
Krzysztof Kaczmarski^1^ and Dariusz Plewczynski^1, 2,*^

Note: All scripts used to generate the results and illustrations in this supplement are available in the GitHub repository: <https://github.com/SFGLab/cudaMMC>.

I. Supplementary Figures

This section provides additional graphical illustrations supporting the findings of the main manuscript.


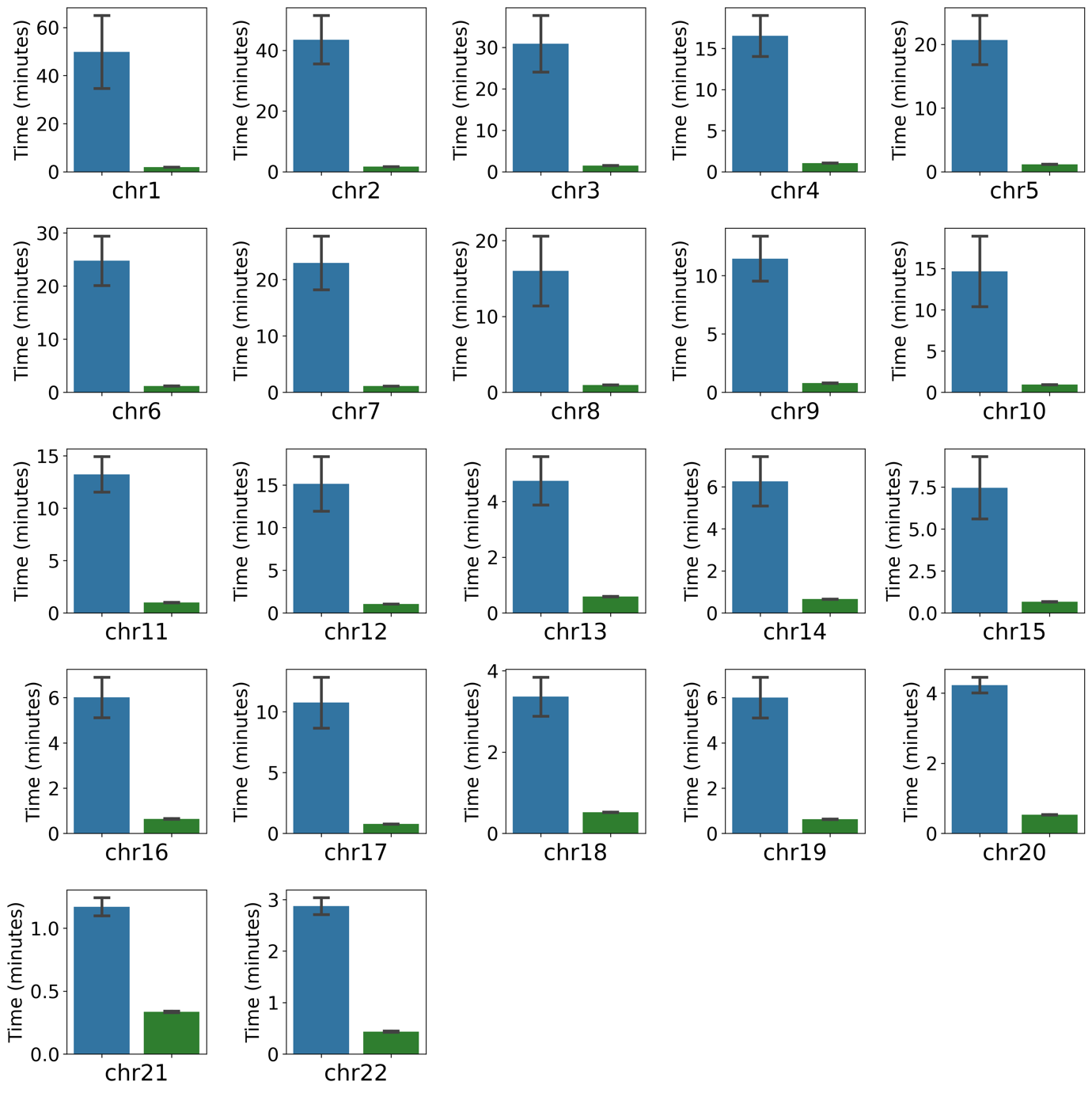


Fig. S1: Time performance comparison for each of the 22 chromosomes. Blue bars represent the 3D-GNOME method and green bars represent the cudaMMC method. Error bars indicate standard deviation. Analysis was performed on data from ST1.


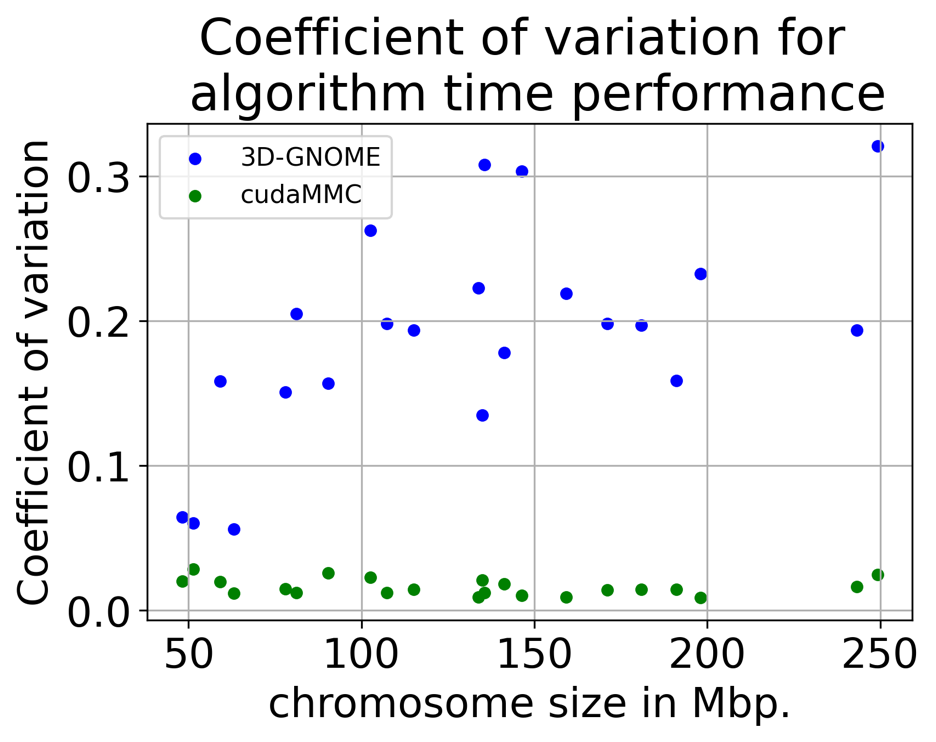


Fig. S2: Coefficient of Variation for algorithm time performance across chromosomes. Blue dots represent 3D-GNOME and green dots indicate cudaMMC. Analysis was performed on data described in ST1.


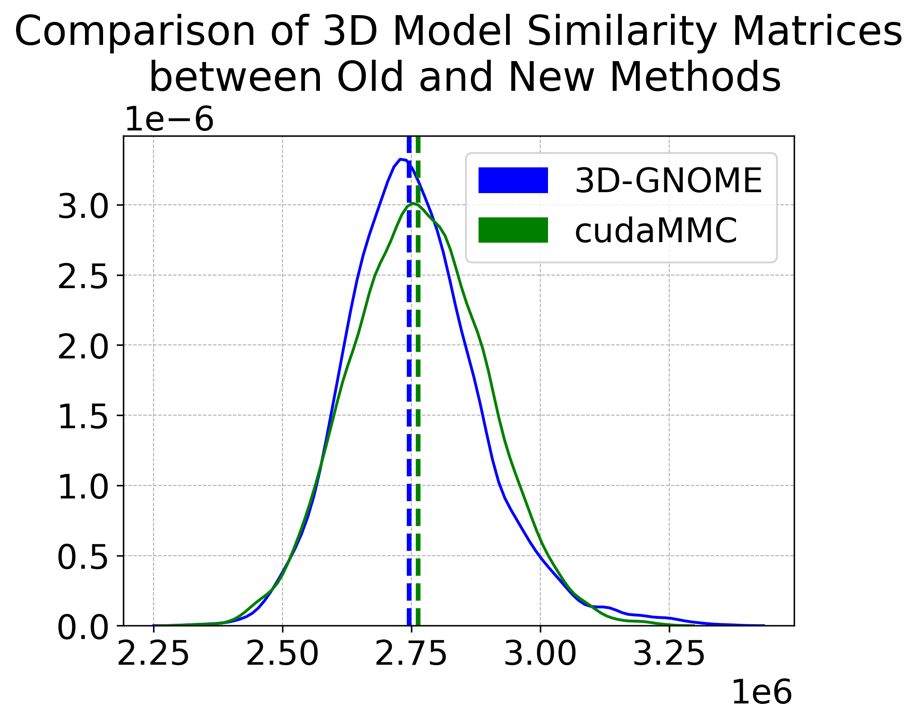


Fig. S3: The distal plot illustrates the 3D model similarity matrices derived from two methods. The horizontal axis displays the similarity matrix values, representing the degree of similarity between models, whereas the vertical axis shows the density of models with specific similarity values. Blue represents models generated by the 3D-GNOME method, while green denotes those from cudaMMC. This analysis was grounded on an ensemble of 100 models produced by each method. Notably, the median difference between the outcomes of the two methods is less than 1%. The approach for this analysis is detailed in the Supplementary Materials of Szalaj et al., 2016."

II. Supplementary Tables

| chrom. | chrom. size (bp.) | PET clusters | singletons | segments | 3D-GNOME (minutes) | cudaMMC (minutes) |
| --- | --- | --- | --- | --- | --- | --- |
| chr1 | 249250621 | 1930 | 2711975 | 380 | 49.84 | 1.92 |
| chr2 | 243199373 | 1526 | 2865208 | 358 | 43.51 | 1.68 |
| chr3 | 198022430 | 1308 | 2312312 | 332 | 30.88 | 1.5 |
| chr4 | 191154276 | 963 | 2144601 | 238 | 16.52 | 1.06 |
| chr5 | 180915260 | 1037 | 1962260 | 266 | 20.67 | 1.16 |
| chr6 | 171115067 | 1227 | 2071017 | 274 | 24.75 | 1.19 |
| chr7 | 159138663 | 1111 | 1580189 | 260 | 22.98 | 1.12 |
| chr8 | 146364022 | 943 | 1468626 | 222 | 16.0 | 0.96 |
| chr9 | 141213431 | 780 | 1030605 | 188 | 11.46 | 0.79 |
| chr10 | 135534747 | 940 | 1223227 | 220 | 14.66 | 0.93 |
| chr11 | 135006516 | 1209 | 1353710 | 216 | 13.23 | 0.98 |
| chr12 | 133851895 | 1111 | 1471015 | 232 | 15.13 | 1.04 |
| chr13 | 115169878 | 448 | 920137 | 140 | 4.74 | 0.59 |
| chr14 | 107349540 | 663 | 827219 | 142 | 6.27 | 0.66 |
| chr15 | 102531392 | 711 | 655278 | 144 | 7.46 | 0.66 |
| chr16 | 90354753 | 693 | 624388 | 134 | 6.01 | 0.64 |
| chr17 | 81195210 | 891 | 786727 | 166 | 10.75 | 0.78 |
| chr18 | 78077248 | 451 | 659029 | 108 | 3.36 | 0.52 |
| chr19 | 59128983 | 712 | 604497 | 114 | 6.0 | 0.63 |
| chr20 | 63025520 | 566 | 495075 | 92 | 4.23 | 0.53 |
| chr21 | 48129895 | 169 | 237111 | 44 | 1.17 | 0.34 |
| chr22 | 51304566 | 387 | 269951 | 64 | 2.87 | 0.44 |

Tab. ST1: Number of interactions used for benchmark modelling with both 3D-GNOME and cudaMMC methods based on long-read ChIA-PET CTCF chromatin interaction data for the GM12878 cell line mapped on GRCh37. Time performance is displayed as the average from 10 modelling runs.

| chrom. | chrom. size (bp.) | PET clusters | singletons | segments |
| --- | --- | --- | --- | --- |
| chr1 | 248956422 | 633097 | 3812238 | 256 |
| chr2 | 242193529 | 589585 | 3953426 | 300 |
| chr3 | 198295559 | 502509 | 3353384 | 248 |
| chr4 | 190214555 | 398075 | 2886478 | 184 |
| chr5 | 181538259 | 419281 | 2819897 | 184 |
| chr6 | 170805979 | 447712 | 2872696 | 200 |
| chr7 | 159345973 | 359657 | 2333672 | 184 |
| chr8 | 145138636 | 327815 | 2227671 | 162 |
| chr9 | 138394717 | 261485 | 1639278 | 148 |
| chr10 | 133797422 | 323205 | 2045009 | 142 |
| chr11 | 135086622 | 346208 | 2045009 | 162 |
| chr12 | 133275309 | 348740 | 2145270 | 150 |
| chr13 | 114364328 | 195844 | 1394545 | 110 |
| chr14 | 107043718 | 226515 | 1352559 | 130 |
| chr15 | 101991189 | 206421 | 1194010 | 108 |
| chr16 | 90338345 | 172906 | 980843 | 110 |
| chr17 | 83257441 | 226504 | 1119489 | 92 |
| chr18 | 80373285 | 162698 | 1100472 | 88 |
| chr19 | 58617616 | 132838 | 629437 | 48 |
| chr20 | 64444167 | 154241 | 897955 | 106 |
| chr21 | 46709983 | 54503 | 343716 | 34 |
| chr22 | 50818468 | 86539 | 427323 | 54 |

Tab. ST2: Number of interactions used for benchmark modelling with the cudaMMC method, based on in situ ChIA-PET CTCF chromatin interaction data for the GM12878 cell line, mapped to GRCh38.

| chrom. | 3D-GNOME ensemble modelling (minutes) | cudaMMC ensemble modelling (minutes) |
| --- | --- | --- |
| chr1 | 4283.69 | 99.33 |
| chr14 | 560.03 | 35.00 |
| chr21 | 84.79 | 10.21 |

*Tab. ST3: Time performance for ensemble modelling of 100 models using 3D-GNOME and cudaMMC, based on long-read ChIA-PET CTCF chromatin interaction data for the GM12878 cell line mapped to GRCh37. Time performance is shown as the average from 3 ensemble modelling runs.*

| Chrom. | cudaMMC modelling on *GRCh38 data* (minutes) |
| --- | --- |
| chr1 | 50.00 |
| chr14 | 16.52 |
| chr21 | 4.33 |

Tab. ST4: Time performance for single modelling using cudaMMC method, based on in situ ChIA-PET CTCF chromatin interaction data for the GM12878 cell line mapped to GRCh38. Time performance is shown as the average from 3 runs.

**References**

Szałaj, P., Tang, Z., Michalski, P., Pietal, M. J., Luo, O. J., Sadowski, M., ... & Plewczynski, D. (2016). An integrated 3-dimensional genome modeling engine for data-driven simulation of spatial genome organization. Genome research, 26(12), 1697-1709.
